# An FPGA Implementation of a Polychronous Spiking Neural Network with Delay Adaptation

**DOI:** 10.3389/fnins.2013.00014

**Published:** 2013-02-13

**Authors:** Runchun Wang, Gregory Cohen, Klaus M. Stiefel, Tara Julia Hamilton, Jonathan Tapson, André van Schaik

**Affiliations:** ^1^Bioelectronics and Neuroscience, The MARCS Institute, University of Western SydneySydney, NSW, Australia

**Keywords:** neuromorphic engineering, polychronous network, time multiplexing, spiking neurons, delay adaptation

## Abstract

We present an FPGA implementation of a re-configurable, polychronous spiking neural network with a large capacity for spatial-temporal patterns. The proposed neural network generates delay paths *de novo*, so that only connections that actually appear in the training patterns will be created. This allows the proposed network to use all the axons (variables) to store information. Spike Timing Dependent Delay Plasticity is used to fine-tune and add dynamics to the network. We use a time multiplexing approach allowing us to achieve 4096 (4k) neurons and up to 1.15 million programmable delay axons on a Virtex 6 FPGA. Test results show that the proposed neural network is capable of successfully recalling more than 95% of all spikes for 96% of the stored patterns. The tests also show that the neural network is robust to noise from random input spikes.

## Introduction

The vast majority of neuromorphic systems developed to date use synaptic weight adaptation to encode information (Boahen, 2006; Indiveri et al., 2006; Serrano-Gotarredona et al., 2009; Giulioni et al., 2011; Pfeil et al., 2012). In this paper we present a system that uses axonal delays to encode and process information.

### Polychronous neural network

Polychronization is the process in which spikes travel down axons with specific delays to arrive at a common target neuron simultaneously and cause it to fire, despite the source neurons firing asynchronously (Izhikevich, 2006). Neural networks based on this principle are referred to as “polychronous” neural networks which are capable of storing and recognizing quite complicate spatial-temporal patterns. Izhikevich (2006) calls these spatio-temporal patterns groups and concludes that “spiking networks with delays have more groups than neurons. Thus, the system has potentially enormous memory capacity and will never run out of groups, which could explain how networks of mere 10^11^ neurons (the size of the human neocortex) could have such a diversity of behavior.” This feature makes a large-scale polychronous spiking neural network an ideal candidate to be used to implement a short-term memory for spatio-temporal patterns. Short-term memory is one of the key parts in cognitive systems.

According to Indiveri and Horiuchi (2011), in their opening editorial for this journal: “One of the Grand Challenges of Neuromorphic Engineering is to demonstrate cognitive systems using hardware neural processing architectures integrated with physical bodies (e.g., humanoid robots) that can solve everyday tasks in real-time.” Our aim is to address this challenge in part by studying the hardware implementation of polychronous networks and the future use of such an implementation as a short-term memory for spatio-temporal patterns in a cognitive neuromorphic system. Here, we report on the first step in this process, i.e., our implementations using FPGAs to implement such a network.

### Spatial-temporal patterns

**Figure 1:**
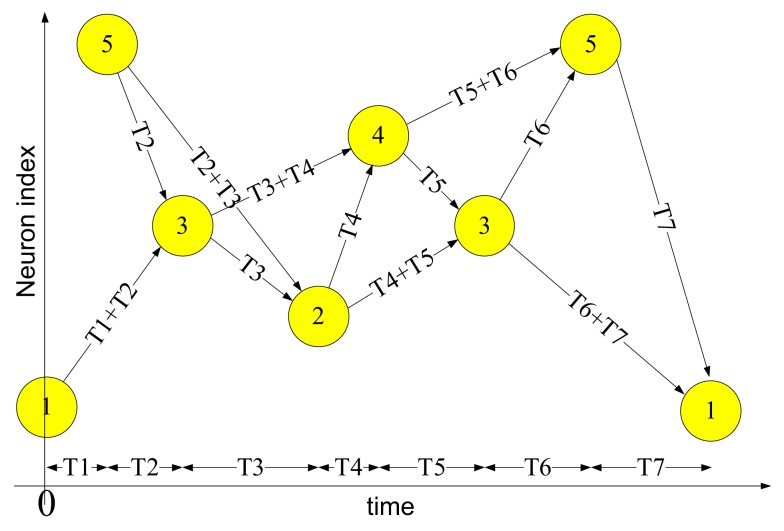
**Example of a spatial-temporal pattern**. The neurons fire asynchronously while their spikes arrive at the destination neurons synchronously, after traveling along axons with appropriate delays. This time-locked relation is the key feature of the spatial-temporal patterns.

Figure 1 shows an example of a spatial-temporal pattern involving five neurons. The threshold voltage of each neuron is set so that it will fire if two pre-synaptic spikes arrive simultaneously. Whenever a neuron fires, its spike is transmitted to all connected neurons via its axonal connections, each of which has its own independent delay. These spikes will then generate post-synaptic currents at the connected neurons.

The example pattern starts when neuron 1 fires at time 0 and neuron 5 fires at time *T*_1_. The spikes from both neurons will arrive at neuron 3 at time *T*_1_ + *T*_2_, and together they will induce neuron 3 to fire at time *T*_1_ + *T*_2_. In the same manner, the spikes from neuron 5 and neuron 3 arrive at neuron 2 simultaneously at time *T*_1_ + *T*_2_ + *T*_3_ and will cause neuron 2 to fire. This process will continue as long as at least two spikes arrive simultaneously at a neuron in the network.

Two procedures are needed to use a polychronous network (such as the one presented above) to memorize and recall spatial-temporal patterns. The first is a training procedure in which the connection delay values of the axon paths between neurons are configured in order to meet the required timing relations of a given pattern. The second is a recall procedure, needed to retrieve a pattern that has been stored in the neural network through training. A pattern can be recalled by presenting the first few spikes of the pattern to the network, after which the network will continue the pattern if it is recognized. For example, to recall the example pattern shown above, neuron 1 needs to fire at time *T*_0_ and neuron 5 needs to fire at time *T*_1_. Together they will cause neuron 3 to fire and the remainder of the pattern will be induced by the network.

The goal of the training procedure is to assign appropriate connection delays to axons in the polychronous neural network so that is able to recall a specific pattern. Generally, this task can be carried out in one of two ways. The first method, which we refer to as *delay selection*, supposes that the delay values of the connections between neurons are random, and fixed at the start of the training procedure. Random stimulation, together with a weight adaptation algorithm, such as Spike Timing Dependent Plasticity (STDP), is applied during training to prune and select appropriate subsets of delays by enhancing the weights of the connections with the desired delays, while decreasing the weights of the undesired delays (Gerstner et al., 1996). The second method, which we call *delay shift*, adapts the actual delay values of the connections between neurons during training. In biology, such adaptation may be achieved by changing the length or thickness of dendrites and axons (Stanford, 1987), the extent of myelination of axons (Stevens et al., 1998), or the density and type of ion channels (Eurich et al., 2000).

Although various algorithms based on *delay selection* have been developed for polychronous networks (Angelini et al., 2007; Paugam-Moisy et al., 2008; Rast et al., 2010; Ranhel et al., 2011), we are not aware of any publication describing a *delay shift* approach for polychronous networks, except our previous work (Wang et al., 2011). Here we present an FPGA implementation of a polychronous network that uses the *delay shift* method.

### Delay shift

We propose two mechanisms to implement the *delay shift* method. The first one is referred to as *delay programming* and the second one is referred to as *delay adaptation*. The delay programming approach relies on a connection storing the delay value between a spike from its input neuron and a spike from its output neuron when both are induced to fire by some external training input. It is not a biologically plausible method, but it is efficient in training and reduces testing time in scenarios where the result will not be affected by the training method. We therefore commonly used it to initialize the network.

**Figure 2:**
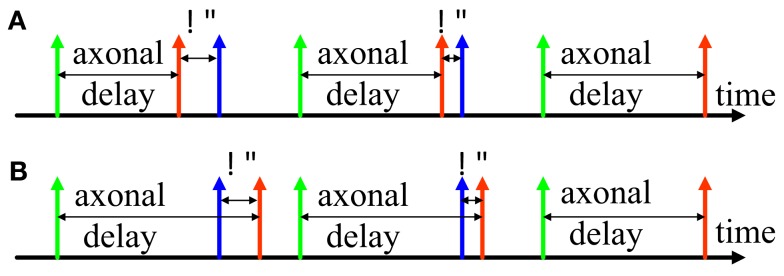
**Delay adaptation**. **(A)** Illustration of delay increment; **(B)** Illustration of delay decrement. The green spike represents a spike generated at the pre-synaptic neuron, the red spike represents the pre-synaptic spike at the synapse, and the blue spike represents the post-synaptic spike at the neuron.

Inspired by STDP, we developed the *delay adaptation* method to fine-tune the delays during the training phase. Two examples of the adaptation of axonal delays are shown in Figure 2, an increment of the delay (Figure 2A) and a decrement of the delay (Figure 2B). After the input neuron fires there is an axonal delay before the pre-synaptic spike arrives at the synapse. If the post-synaptic spike, induced by other neurons in the network or by external input, is not simultaneous with the pre-synaptic spike at the synapse, we may adapt the axonal delay by increasing or decreasing it by a small amount. This procedure is repeated until the pre-synaptic spike at the synapse occurs simultaneously with the post-synaptic spike. In the later part of this paper, we will present three different strategies for *delay adaptation* that use different methods to determine the step size of the increments or decrements.

In the training phase, delay adaptation causes the connections to attain the desired delays through repeated presentation of the desired spatial-temporal patterns. The *delay programming* method can be regarded as a special case of the *delay adaptation* method in which the delay adaptation is completed in just a single step and never altered. With the *delay adaptation* method, every time a pattern is recalled the delay values in the pattern will be updated, allowing the learned delays to be modified.

## Materials and Methods

### Digital implementation of spiking neural networks

To date, all the published polychronous spiking neural networks have been implemented using software neuron models [with the exception of our previous work (Wang et al., 2011)] and such simulations are not capable of achieving real-time emulation of large-scale neural networks. In Izhikevich (2006), the author presented that “the small size of the system does not allow us to explore other cognitive functions of spiking networks… One second of simulation took more than 1 month on a cluster of 27 3-GHz processors.” In our previous work we described an analog implementation capable of providing real-time emulation, but it was limited to a small size (14 neurons and 364 programmable delay axons). A digital FPGA solution was chosen over an analog implementation due to the relative ease in developing and debugging such a system. Modern FPGAs provide a large number of logic gates and physical memory, allowing large-scale neural networks to be created at a low cost. Unfortunately, such a solution may lead to a loss of biological plausibility.

There are three main types of digital implementations for large-scale neural networks; hardware-based designs, customized microprocessor systems, and networks built using conventional microprocessors. Hardware-based designs, such as the one presented here, use the standard ASIC/FPGA design flow and include such examples as the EU SCANDLE project, which has 1M neurons (Cassidy et al., 2011) and the SyNAPSE project which consists of 256 neurons and 65,000 plastic synapses (Merolla et al., 2011; Seo et al., 2011). Hybrid microprocessor/hardware systems are another means of implementing a digital neural network. Hardware functional models (similar to hardware accelerators) are used to generate data, and traditional microprocessors are employed for further processing. An example of such a system is the STDP implementation by Belhadj et al. (2008). Digital neural networks can also be implemented using large networks of embedded microprocessors, such as in the spiNNaker project (Rast et al., 2010) in which ARM processors, running software neuron models, perform the calculations instead of physical silicon neurons. These systems are essentially generalized networks that have been highly optimized for neuron computation.

### Proposed polychronous network

#### Neural network

**Figure 3:**
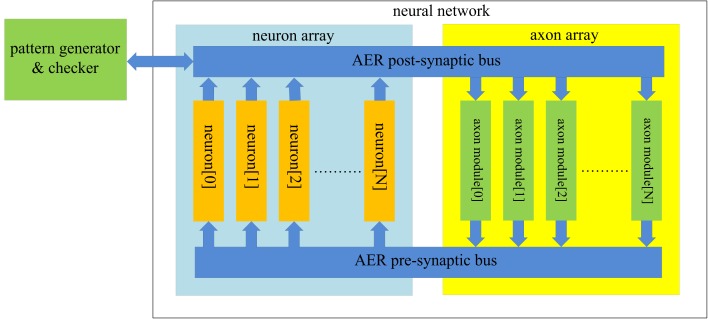
**Structure of the proposed polychronous neural network**. The neuron array generates post-synaptic spikes and then sends them to the axon array, which propagates these post-synaptic spikes, with programmable axonal delays, and generates the pre-synaptic spikes at the end of the axons. These pre-synaptic spikes are sent to the neuron array to cause the neurons to fire. The connectivity and delay of the axons in the axon array are all configurable.

The structure of the proposed neural network is shown in Figure 3. It contains two functional modules: a “neuron array” and an “axon array.” The neurons and the axon modules communicate with each other via Address-Event Representation (AER) buses (Boahen, 2000).

Each neuron in the neuron array is identical in structure and has a unique AER address. The axon modules in the axon array are also identical in structure, and have both unique and configurable addresses. The axon modules generate pre-synaptic spikes, which are received by the neurons. The neurons will then generate post-synaptic spikes if more than a certain number of pre-synaptic spikes arrive simultaneously (see Figure 1). In the example of a spatial-temporal pattern shown in Figure 1, two simultaneous pre-synaptic spikes arrive close enough to cause each subsequent neuron to fire. In the actual implementation, we avoid coincidence detectors with only two input since they are easily affected by cross-talk caused by the overlap of spatial-temporal patterns. To decrease the likelihood of such cross-talk between patterns, we used coincidence detectors with four inputs and a threshold of three spikes.

The post-synaptic spikes are sent to the axon modules in the axon array, which propagates these post-synaptic spikes with axonal-specific delay values and generates pre-synaptic spikes at the end of the axons. In the proposed neural network, the communication between any two neurons must be conducted via the axon modules in order to implement the polychronous network. There is an additional module in Figure 3 labeled as the “pattern generator and checker,” which is an external module used to perform the training and recalling functions.

#### AER bus

The AER bus and protocol used in this system differs slightly from the standard AER bus and protocol (Boahen, 2000). We do not use handshaking, so we have omitted the request and acknowledge signals. Instead we use “active” lines to tell the receiver (neurons or axon modules) that a spike has been placed on the bus. Since we are addressing the arrays as a linear array, i.e., with a single binary address, as opposed to the row/column addressing with two binary addresses used in a standard AER bus (Boahen, 2000), our bus tends to be wider. The width of the address bus is decided by the size of the neuron array. For instance, if the neuron array has 4096 neurons, the address bus comprises 12 bits with an address range from 0x000 to 0xFFF. Without the active line, a 12 input OR gate would be needed in every module to check if an address is present on the bus.

**Figure 4:**
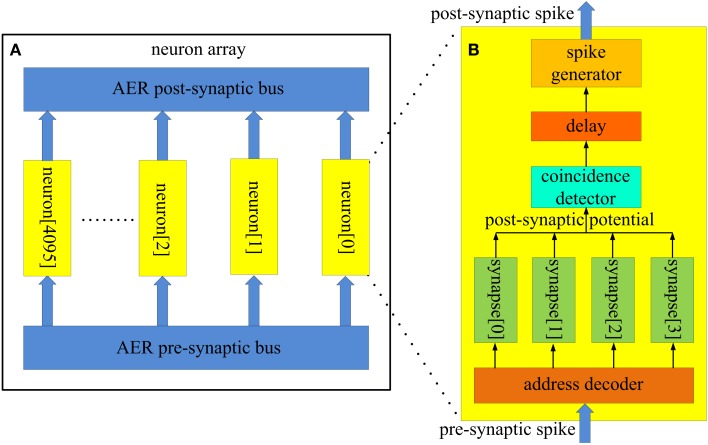
**Structure of (A) the neuron array; (B) the coincidence detector neuron**. About 4k identical coincidence detector neurons (each with a unique address) are placed parallel in the neuron array. These neurons receive pre-synaptic spikes from the axon modules in the axon array via the AER pre-synaptic bus. They generate post-synaptic spikes and send these spikes to the axon modules in the axon array via the AER post-synaptic bus. The neurons are used as coincidence detectors (see Figure 1). If three out of four pre-synaptic spikes (with the same address) arrives within a time window (e.g., 1 ms), the coincidence detector neuron, the address of which matches the address of these spikes, generates a post-synaptic spike.

Two different AER buses are used in our spiking neural network. The first one is referred to as the AER post-synaptic bus, and is used to transmit post-synaptic spikes generated by the neurons to the axons. It uses a single active line in addition to the binary address of the event. The pattern generator and checker module is also connected to this bus for training and recalling purposes. The second AER bus is referred to as the AER pre-synaptic bus and it is used to transmit pre-synaptic spikes generated by the axon modules to the neurons (see Figure 4). Each neuron receives input from four neurons via four axons in our network. The pre-synaptic bus therefore uses four active lines, one for each synapse of the neuron.

A further difference in our AER implementation is that there is no arbiter to deal with collisions when two addresses are placed on the bus simultaneously. Not having an arbiter reduces hardware costs, but a collision will generate an incorrect address on the bus and the spikes will be sent to the wrong receiver. It might seem counterintuitive to not have an arbiter in a neural network that aims to detect coincidences. However, spikes on the bus will only last for less than a microsecond, while the time window in which the four spikes in our polychronous network are considered coincident is 1 ms long. Spikes will therefore be unlikely to be exactly synchronous and collisions will be rare. When they happen, they can be viewed as a type of noise that the polychronous network is relatively robust against. We will present further details on how we deal with this in the final paragraphs of the Section “Time-Multiplexed Axon Array,” once we have presented the detailed FPGA implementation of the axon array to the reader.

#### Neuron array

Figure 4A shows the structure of the neuron array: a number of identical neurons (each with its own address) are placed in parallel in the array and share one input bus (the AER pre-synaptic bus) and one output bus (the AER post-synaptic bus). The size of the neuron array is a trade-off between performance and hardware cost. We have implemented six different sizes of neuron arrays, ranging from 128 to 4096 (4k) neurons. The performance for different sizes will be presented in the results section. In this paper we use a network consisting of 4k neurons for illustrative purposes.

The neurons in the neuron array work as coincidence detectors that detect how many pre-synaptic spikes have arrived simultaneously. Figure 4B shows the structure of the coincidence detector neurons. It has one address comparator, four synapses, one coincidence detector, one delay module, and one spike generator. The address comparator compares the address of the incoming pre-synaptic spikes with the address of the neuron. The four active lines control which synapse will be used to generate a post-synaptic potential which is then sent to the coincidence detector.

The coincidence detector will generate a coincidence signal if at least three of the most recent four inputs are coincident. This coincidence signal is delayed for a certain amount of time, which simulates the neural delay between synaptic inputs and the post-synaptic output spike, and is then sent to the spike generator, which will generate a post-synaptic spike and place its address on the AER bus.

#### Axon array

The structure of the axon array is shown in Figure 3. It is very similar to the structure of the neuron array as it consists of a number of identical axon modules placed in parallel. The proposed axon array is capable of achieving much higher resource utilization than the axon-select method used in our previous work (Wang et al., 2011). In that work, we proposed a supervised learning rule that generates spatial-temporal patterns based on a fixed connectivity between neurons. The conclusion from that work was that there were always some axonal delay paths that remain unused. To utilize all axonal delay paths we switch our strategy from selecting existing axonal delay paths to generating delay paths *de novo*, so that only connections that actually appear in the training patterns will be created. Additionally we configured the system such that there can be any number of axonal delay paths between any two neurons in the network.

**Figure 5:**
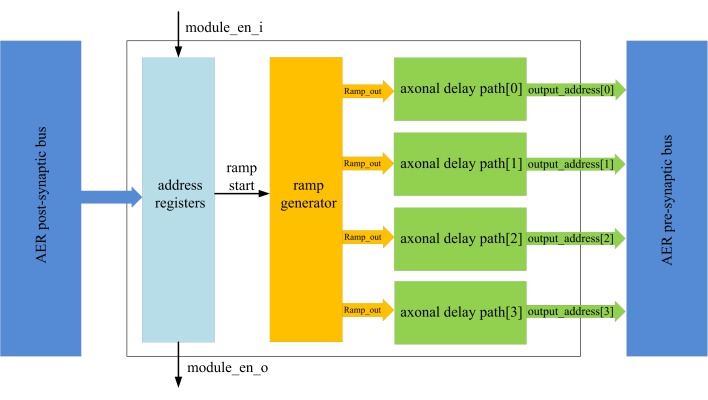
**Structure of the axon module**. The axon module receives post-synaptic spikes generated by the neuron in the neuron array via the AER post-synaptic bus. The axon module propagates these spikes with axonal-specific programmable delays and generates pre-synaptic spikes at the end of the axons. The address registers are used to store the input address and the four output addresses for the axonal delay paths.

Figure 5 shows the structure of the axon module: it has five address registers, one ramp generator, and four identical axonal delay paths. The address registers are used to store the input address and the four output addresses for the axonal delay paths. To place one axon module between neurons, we need to configure its address registers. The ramp generator will generate a digital count that increases linearly with time after a reset by a start signal. This digital axonal delay carries out delay programming and delay adaptation in the same manner as the analog VLSI programmable axonal delay in (Wang et al., 2012).

At the beginning of the training, axon module[0] (see Figure 3) is enabled and all the other axon modules are disabled. When the first post-synaptic spike in a training pattern arrives, axon module[0] will latch the address of this spike as its input address and enable axon module[1]. Each axon module will configure its addresses (input address and output addresses) only once, unless a full system reset is performed. The output addresses will be configured after the input address is configured. As there are four output addresses, one for each of the destination neurons, it will take four iterations for one axon module to finish the configuration of its output addresses (using the addresses of the next four sequential post-synaptic spikes in the training pattern after its input address is configured).

The delay programming is carried out in the same way as the address configuration. At the beginning of the training, when the first post-synaptic spike arrives at axon module[0], it will reset the ramp generator and begin counting. The ramp generator will send the value of the counter (ramp_out) to the four axonal delay paths. The delay of each axonal delay path is programmed (referred to as ramp_latched) when the output addresses are being configured (when the next four sequential post-synaptic spikes from the training pattern arrive) and it will not be programmed again until after a reset.

After the delay programming, when a post-synaptic spike arrives and its address matches the input address of one axon module, the ramp generator will start counting again. The axonal delay path will compare the output of the ramp generator with the programmed delay. A pre-synaptic spike will be generated when the output of the ramp generator exceeds the programmed delay with an address stored in the output address register.

The delays can also be configured using delay adaptation rather than delay programming. In the delay adaptation algorithm the axonal delay is increased or decreased based on the delay between pre-synaptic spike and post-synaptic spike (see Introduction). In this mode, the axonal delay path needs to do two things: measure the firing time of these two spikes and calculate the difference between them; and adapt the axonal delay stored in the latch.

To calculate the time difference, we need to know when the pre-synaptic spike arrives, and when the post-synaptic neuron fires. The time at which the neuron fires is represented by the output of the ramp generator. The time at which the pre-synaptic spike arrives is represented by the programmed axonal delay stored in the latch. By comparing these two values, the delay can be obtained. One major advantage of this structure is that all these variables are local (in one axon module) and they can be adapted without affecting the other axon modules.

There are three strategies for altering the delay value of each axon. The first strategy is to correct the delay to the desired value in one step, simply changing the value of the programmed delay to the value of the ramp generator. This case is similar to the delay-programming mode. The second strategy is to change the value of the programmed delay by a single binary count. The last strategy is to change the delay proportionally to the calculated time difference. This could be a linear proportion (i.e., multiplying by a coefficient) or a non-linear proportion (such as an exponential relation).

We have implemented all three strategies. The first method is identical to just using the delay-programming method and the second method, which uses a single step, is impracticably slow and produces similar results to the third method with a coefficient of 0.5. Therefore only the results obtained by using the third strategy will be presented and discussed in Section “Results.”

### FPGA implementation

This section will focus on the implementation of the whole system including the proposed neural network, the “Pattern generator and checker,” and the debug block. We will also present how all these blocks are integrated. The system was implemented using Verilog HDL on a Virtex 6 FPGA with the system clock running at 66 MHz.

#### Time-multiplexed axon array

An implementation that directly maps all the axon modules to hardware is too expensive and will limit the size of the axon array. As this system is designed for real-time emulation, there is no requirement for a high time resolution. Typical axonal delays in mammalian cortex range from 0.1 to 44 ms (Swadlow, 1985, 1994), meaning that a resolution higher than 100 μs will be enough for emulating biological axons. The system clock runs at 66 MHz and 100 μs × 66 MHz = 6600 which is the maximum clock cycles we have within 100 μs.

**Figure 6:**
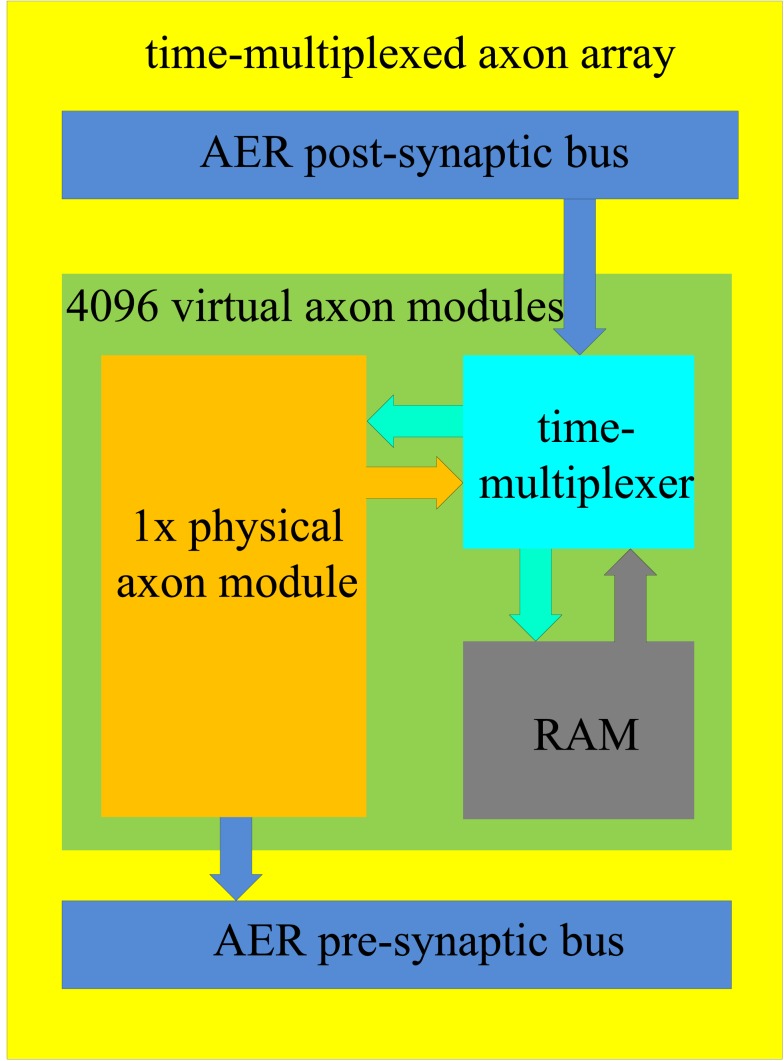
**Structure of the time-multiplexed axon array**. The physical axon module computes the data for one virtual axon module in one clock cycle. The time-multiplexer provides the physical axon array with its input data, which is read from the RAM, and writes the computed output data to the RAM in one clock cycle.

We propose a time-multiplexed axon array which comprises 4096 virtual axon modules (see Figure 6). It has one physical axon module, a RAM, and a time-multiplexer. The structure of the physical axon module is identical to the structure described previously (Figure 5). All the values (such as the addresses, the programmable delay, and so on) of the physical neuron are stored in the RAM, similar to the way a general CPU works. The time-multiplexer processes the virtual axon modules sequentially. For any given virtual axon module, the time between two operations will take 4096/66 MHz ≈ 62 μs which is still smaller than the 100 μs resolution desired.

**Figure 7:**
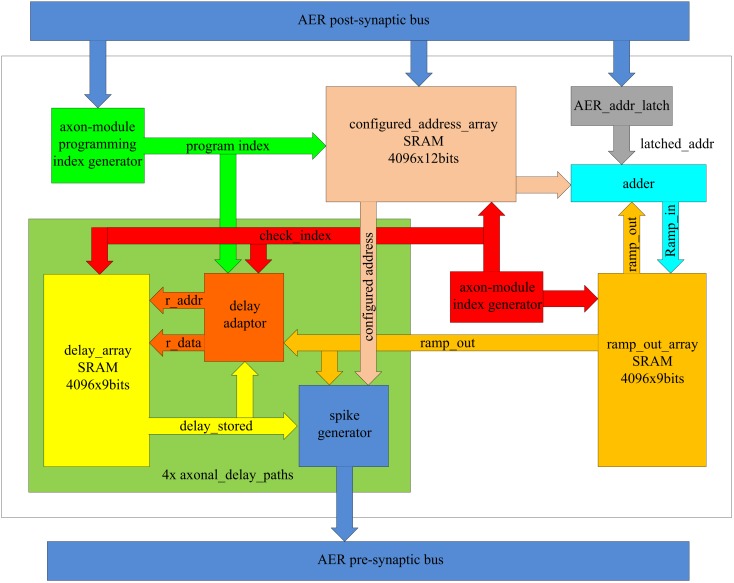
**Schematic of the time-multiplexed axon array**.

Figure 7 shows the time-multiplexed axon array in more detail. Each axon contains four identical axonal delay paths, a programming index generator, an axon-module index generator, an SRAM for the configured address (configured_address_array), a register to hold the current address of the input spike (AER_addr_latch), and an adder. The axonal delay paths each contain a delay adaptor, a spike generator, and an SRAM to store the output of the ramp generator (ramp_out).

The axon-module index generator is a central component as it informs other components as to which virtual axon module is currently being computed. It is composed of a 12-bit counter, with the value of this counter being referred to as the axon-module index. After power up, the axon-module index is reset to 0x000 and is incremented by one on each clock cycle.

The axon-module programming index generator informs the other components as to which virtual axon module is to be configured, and also consists of a 12-bit counter. After power up, it is initialized to 0x000 and it is increased by 1-bit whenever a post-synaptic spike arrives, until it reaches its maximum value (0xFFF). When this value is reached, all the virtual axon modules will have been used and no further configuration for addresses and delay programming is possible.

The configured address array is used to store addresses and is composed of one dual-port SRAM with a memory depth of 4096 and a width of 12 bits. Each axon module has one input address and four output addresses. Each of these four output addresses is identical to one of the input address of each of the next four axon modules, respectively, and therefore only one address register is needed for each virtual axon module.

During training, the address of an arriving post-synaptic spike is written into the configured address array, with the programming index used as the writing address. The output port will send out the contents on every clock cycle (as virtual axon modules are evaluated cycle-by-cycle). For simplicity, the timing specifications related to the memory are not presented. Whenever a post-synaptic spike arrives during a recall procedure, the AER_addr_latch register will hold the address of this spike for the next 4096 clock cycles (one clock cycle for each virtual axon), before clearing it.

The system uses a comparator to check if the latched address matches the configured addresses of each virtual axon module. If a match is detected, the ramp generator of that virtual axon module is started. The adder increments the output of the ramp generator serially on each clock cycle until it reaches its maximum value. Multiple address matches are possible as more than one axon module may be configured with the same address as a neuron may appear in different positions in one or more patterns.

The axonal delay path stores the delay value and generates a pre-synaptic spike. It comprises an SRAM (delay_array) to store the delay, a spike generator, and a delay adaptor. It is instantiated four times in hardware on the FPGA to create four identical copies. The axonal delay path can be used to implement both the delay-adaptation mode and the delay-programming mode.

When using delay programming, the delay adaptor is bypassed and the output of the ramp generator is sent directly to the SRAM as input along with the programming index. Whenever a post-synaptic spike arrives, the output of the ramp generator is written into the SRAM at the memory address computed from the programming index.

When using delay adaptation, the delay adaptor will initialize each stored delay with a random value. The delay adaptor calculates the difference between the output of the ramp generator and the programmed axonal delay (see *Axon module*). If the value is not zero, the delay stored will be adapted according to one of the three strategies mentioned above. Delay adaptation is carried out for each virtual axon module serially and cyclically.

All the SRAM used in the axon is composed of block RAM built into the FPGA and in order to achieve maximum capacity (for storing spatial-temporal patterns), the utilization of this hardware resource needs to be maximized. We chose a memory with a size of 4k × 9 bits = 36k bits, as the size of the on-chip block RAM in the Xilinx Virtex 6 family is either 18 or 36k. Any block memory used has to be constructed from RAMs of these two sizes and any fragments are wasted. To fully utilize the available memory, an implementation with 4096 virtual axons was chosen.

Each virtual axon module is updated every 62 μs (4096/66 MHz), and therefore the longest delay the ramp generator can generate is 2^9^ × 4096/66 MHz ≈ 32 ms. This value can be easily changed if desired, by either changing the clock frequency or the bit width of the counter.

If the output of the ramp generator exceeds the programmed delay, a pre-synaptic spike will be generated with the AER address stored in the SRAM for the configured address. This programmed delay corresponds to one of the four axonal delay paths in each virtual axon module. To achieve a reliable commutation, the pulse width of this pre-synaptic spike is set to 16 clock cycles and during that period no other pre-synaptic spike will be generated, as it would generate an incorrect address. In other words, a theoretical maximum of 4096/16 = 256 axon modules can be active during any 62 μs cycle for this configuration.

As mentioned previously (see AER Bus), not having an arbiter reduces hardware costs, but a collision will generate an incorrect address on the bus and a spike will be sent to the wrong receiver. To increase the possibility that the spikes are sent to the correct receivers, we increase the pulse width of the spikes. So even if there is a collision on the AER bus, it will most likely not be for the full 16 clock cycles, so that spikes can still be sent to the correct receivers at the start and end of the collision. For the duration of the overlap, a spike will indeed be sent to a wrong address, but its effect will quickly die out in the system when it is not part of a learned set of time-locked relations. This is a feature typical of a polychronous spiking neural network. The more clock cycles a spike lasts, the more robust the communication will be. On other hand, the more clock cycles a spike lasts, the larger the chance of a collision. For flexibility, the pulse width is designed to be configurable from 1 to 16 clock cycles. Our test results show that a pulse width of four clock or more clock cycles provides reliable communication. More details will be presented in Section “Results.”

#### Multiplexed neuron array

As was the case for the axon array, a full physical implementation of the neuron array would be too expensive in terms of hardware. Time multiplexing could also be used to implement the neuron array using the same topology as the time-multiplexed axon array shown in Figure 6. A physical neuron takes the place of the physical axon module and, whereas the axon array uses the axon-module index generator to control the virtual axon modules, the neuron array uses a neuron index generator to control the virtual neurons.

This time-multiplexed neuron array works well when the probability of pre-synaptic spikes arriving at two different neurons within the 62 μs update cycle is low, i.e., the total number of axons in the network is low. When a pre-synaptic spike is generated by the axon array, it will take the time-multiplexed neuron 4k clock cycles (62 μs) to check if this spike is destined for any of the 4k virtual neurons before it can process another spike from the axon array. If another spike arrives during this period, it will be dropped, which would lead to a decrease in performance of the neural network, as measured by successful recall of stored patterns. The more patterns that are stored in the network, the more axons are needed, and the more likely simultaneous spikes become. In our tests, we found that the network would start performing poorly when the number of virtual axons became larger than four times the number of virtual neurons.

To implement networks with many more axons than neurons, we propose a structure with several physical neurons that are then time-multiplexed. We found that only a small percentage (less than 5%) of the neurons in the neural array are active at any given time, so only a small number of physical neurons need to be implemented (e.g., 128 physical neurons for a 4k neuron array).

**Figure 8:**
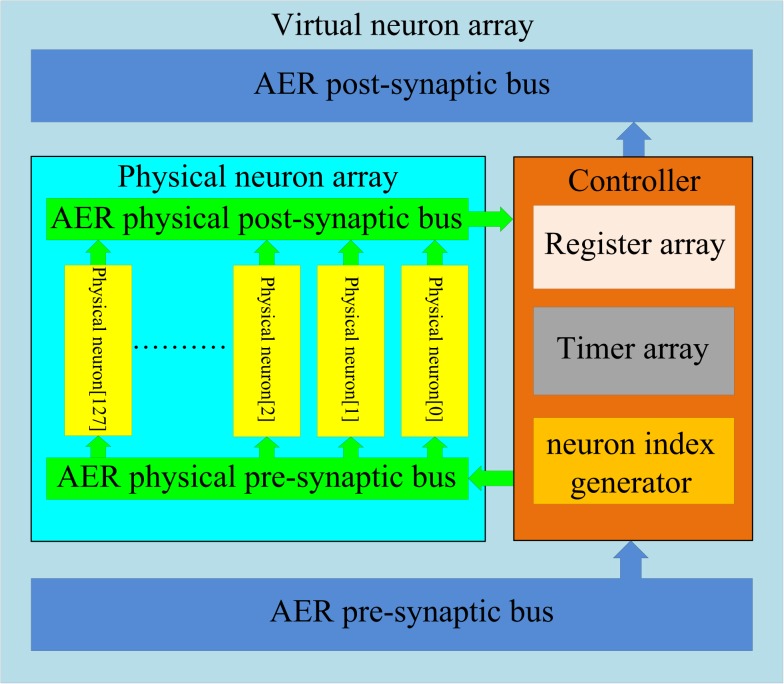
**Structure of the multiplexed neuron array**. In the multiplexed neuron array, 4k virtual neurons are achieved by using 128 physical neurons and one controller. The controller sends the pre-synaptic spikes to the physical neurons depending on the usage of the physical neurons. The controller receives the spikes generated by the physical neurons and sends these spikes (with a remapped address) to the axon modules in the axon array.

The structure of the multiplexed neuron array is shown in Figure 8. It consists of two sub-blocks: a physical neuron array and a controller. They communicate with each other via two internal AER buses: the AER physical pre-synaptic bus and the AER physical post-synaptic bus. The controller receives pre-synaptic spikes from the axon array and assigns them to the physical neurons for the generation of post-synaptic spikes which will be sent to the axon array. From the point of view of the axon array, the multiplexed neuron array appears as a neuron array with 4096 neurons.

##### Physical neuron array

The physical neuron array can be regarded as a miniature version of the neuron array shown in Figure 3 as it has the same topology: 128 identical neurons (each with its own address) are placed in parallel in the array and share one input bus (AER physical pre-synaptic bus) and one output bus (AER physical post-synaptic bus).

The number of physical neurons needed in the array depends on the number of axons in the neural network. The more virtual axons, the more physical neurons are needed. We have implemented various sizes of the physical neuron array ranging from 16, 64, 128, and 256 neurons (to ensure a full utilization of the AER bus, it should be a power of 2). The results showed that the size of 128 is large enough without missing any pre-synaptic spikes even at the highest firing rate. We will use this configuration for illustration.

The physical neuron is implemented using a coincidence detector that generates a post-synaptic spike when three or more pre-synaptic spikes arrive within 1 ms of each other. After generating a post-synaptic spike, a 1 ms refractory period is employed to prevent the generation of multiple post-synaptic spikes for a single event, and to better emulate biological behavior.

**Figure 9:**
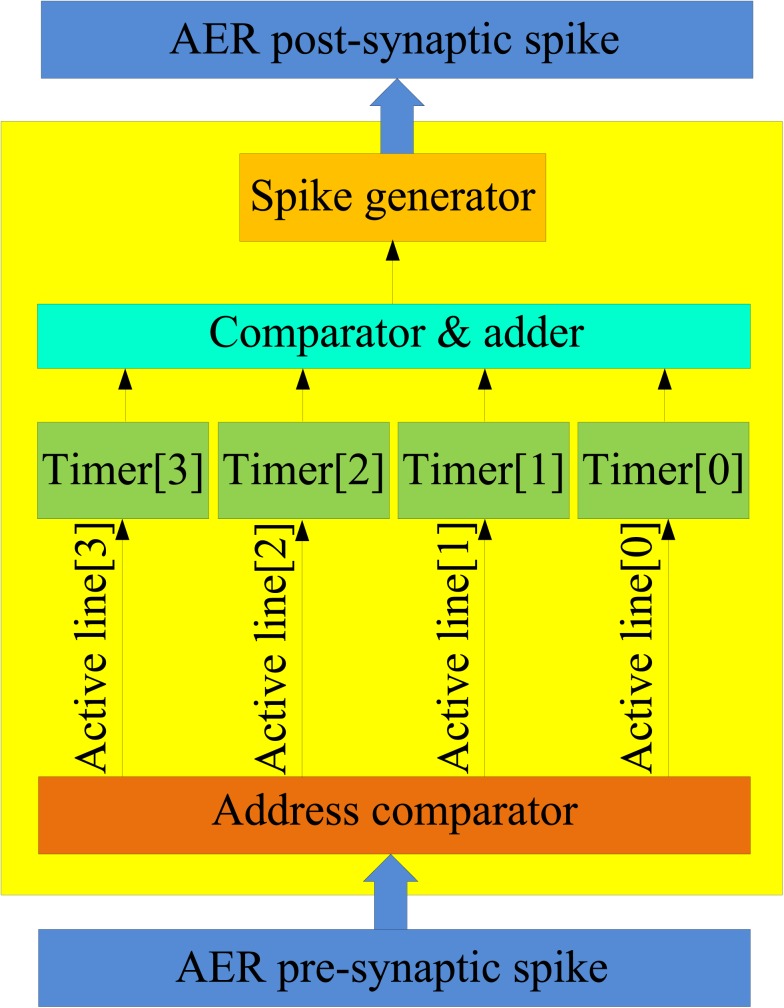
**Structure of the physical neuron**. The physical neuron is implemented using a coincidence detector that generates a post-synaptic spike when three or more pre-synaptic spikes arrive within 1 ms of each other.

Figure 9 shows the structure of the physical neuron. When a spike arrives from the AER pre-synaptic spike bus at one of the physical neuron’s four synapses, the corresponding 1 ms timer is activated. (Note that the virtual neurons can have many more than four synapses.) If that timer is already active, then the spike is simply ignored. The four timers are needed so that three spikes that are not exactly simultaneous, but that arrive within 1 ms of each other can still generate an output spike via the comparator. The output spike generation will be delayed from the time that the third spike arrives by a certain amount. This delay is used to emulate the integration time of a real (biological) coincidence detecting neuron. In such a neuron, the delay between the generation of a spike and the arrival of the pre-synaptic spikes is a function of the temporal dispersion of these pre-synaptic spikes. For instance, if all four pre-synaptic spikes arrive simultaneously, the integration time will be relatively small, while if only three pre-synaptic spikes arrive within 1 ms with two 500 μs spike intervals, then the integration time would be longer.

Rather than implementing real integration of pre-synaptic inputs, which would use a significant amount of hardware resources in a digital implementation, we use the sum of the values of the timers when the third spike arrives and delay the generation of the post-synaptic spike by this amount.

##### Controller

The controller comprises three components: a register array, a timer array, and a neuron index generator. The register array contains 128 12-bit registers that store the addresses of the pre-synaptic spikes (referred to as virtual address) from the axon array. The timer array comprises 128 identical 1 ms timers. Each physical neuron is linked to one register and one timer. These two arrays are controlled by the neuron index generator, which is composed of a 7-bit counter. It plays a similar role to that of the axon index generator in the time-multiplexed axon array. The axon index generator controls the virtual axon modules, whereas the neuron index generator controls the physical neurons (as well as the linked registers and timers). The output value of this neuron index generator is used as the address of the physical neurons.

As mentioned before, the controller dynamically assigns physical neurons to each incoming pre-synaptic spike. The physical neurons, which are coincidence detectors, are used to detect how many pre-synaptic spikes have arrived within 1 ms of each other. When a spike arrives from the axon array and a physical neuron has been assigned for the arriving spike’s address, the spike will be sent to that neuron. Otherwise it will be sent to an unassigned neuron, which will then be labeled as assigned by the controller. Once the timer of that neuron has expired (after 1 ms), the neuron will be freed and labeled as unassigned by the controller.

The neuron index generator compares the address of any newly arriving pre-synaptic spike with the values (stored addresses) in the register array concurrently. If a match is detected and the timer linked to that register is active, this indicates that the linked neuron has been assigned for coincidence detection. The controller will then send this spike to that neuron. If no match is detected, the controller will carry out the following actions simultaneously:

1. Store the address of this pre-synaptic spike in the register indicated by the current value of the neuron index generator.
2. Start the timer linked to that register.
3. Send this spike to the linked neuron.

After finishing the above actions, the neuron index generator is incremented by one. It will count around to zero after it reaches the maximum value. When a post-synaptic spike is generated by the physical neuron, the controller will send it to the axon array with an address which is stored in the register linked to that physical neuron.

#### Pattern generator and checker

The pattern generator and checker module generates spatial-temporal patterns for training and for testing whether the patterns are recalled successfully during recall. To create random spatial-temporal patterns, we need to assign each spike in the temporal sequence to a random selected neuron in the network. This is implemented using two Linear Feedback Shift Registers (LFSRs), which generate two pseudo-random numbers. The first generated number is used as the index of the next neuron in the sequence to generate the spike after an inter-spike interval from the previous spike in the sequence determined by the second pseudo-random number.

Identical patterns are generated for both training and recall based on the seed of the LFSRs. During training, the entire pattern is sent to the neural network. During recall, only the initial segment, comprising the first four spikes of each pattern, is sent to the polychronous network. The rest of the pattern generated by the LFSRs is compared with the pattern retrieved from the neural network. This comparison is carried out in a similar way as in the coincidence detector in the physical neuron. The pattern checker generates a 4 ms pulse for each spike generated by the pattern generator. To increase the dynamics of the system, each pulse is generated earlier than the generation of a spike with a random offset which ranges from 500 μs to 1 ms. A coincidence is recorded when a spike arrives from the neural network during that pulse and the addresses match. At the end of the pattern, the comparator checks how many coincident spikes have been recorded. If this number is larger than 70% of the pattern length, we define this pattern as having been recalled successfully. This threshold is configurable and we use the value 70% here for illustrative purposes only. In the Section “Results” we investigate the number of patterns recalled successfully for various thresholds.

The pattern generator can also create a noise input (random spikes) for the network using a third LFSR. After power up, it continues to generate random spikes, which follow a Poisson distribution according to a configurable firing rate (Linares-Barranco et al., 2007). The random number generated by this third LFSR is not only used as the inter-spike interval, but is also used as the index of the next random neuron to fire. These random spikes may be sent to the axon array via the AER post-synaptic bus. The effect of the noise at different firing rate will also be presented in the Section “Results.”

#### System integration

**Figure 10:**
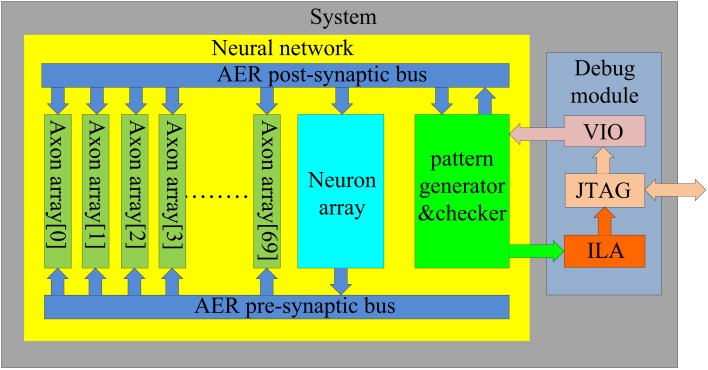
**Schematic of the full system**. To achieve the maximum utilization of the hardware resources, seventy axon arrays are placed parallel in the system. During the training procedure, they are trained serially, while in recalling mode, they work in parallel.

Figure 10 shows the structure of the full system. We can incorporate more than one axon array into the system in order to increase capacity. Each axon array is connected in parallel in the neural network (see Figure 10). Each axon array works with the neuron array as presented in the Section “Neural Network.”

At the start of training, the first axon array (axon array[0]), is enabled for configuration and all the others are disabled. When all the axon modules in axon array[0] have been configured, the axon array[1], is enabled for configuration. The procedure repeats until all the axon arrays are configured. In this way, all the axons in all the axon arrays can be used in the proposed system. We integrated 70 axon arrays in the system, yielding 4096 × 4 × 70 ≈ 1.15M axons (with variable delays) in total. Table 1 shows the utilization of the hardware resources on the FPGA for this configuration.

**Table 1:** **Device utilization Xilinx Virtex XC6VLX240T**.

Resource	Used	Total available
Occupied slices	36,994	37,680
Slice FF’s	58,341	301,440
Slice LUTs	134,665	152,720
LUTs as logic	91,101	152,720
LUTs as RAM	42,420	58,400
36K RAMs	402	416

The debug module (Figure 10) contains a virtual input/output (VIO), an integrated logic analyzer (ILA), and a JTAG interface. These are IP cores provided by Xilinx and can be integrated into the design. The VIO and ILA are controlled through the JTAG interface and allow access the internal signals on the FPGA. In our system, the VIO is used to send parameters to the pattern generator and the ILA is used to collect the test results. These two IP cores greatly simplify the testing process, as the tests for different scenarios can be created easily by altering parameters and retrieving the results via the JTAG interface.

The entire system was designed using the Xilinx design tools with exception of the simulation, which was performed using the Cadence NC-Verilog tool. A bottom-up design flow was adopted in which we designed and verified each module separately. Once the module level verification was complete, all the modules were integrated together for chip-level verification. The Xilinx tool was then used for synthesis and implementation and the Chip Scope tool for testing.

## Results

The results obtained from the neural network presented above will be discussed in this section. The results are organized in five sections: delay programming, delay adaptation, characterization of pattern recall, effect of noise, and FPGA capacity testing. This is followed by a Section “Discussion.”

### Delay programming

In the setup for the delay-programming tests, a single axon array was used in the neural network, yielding 4k axon modules with 16k (16384) axonal delay paths (variables). Note that unlike in Izhikevich (2006), no connections are shared between two patterns, so that the number of available connections directly determines the maximum number of inter-spike intervals that can be programmed into our network. As each inter-spike interval in a pattern consumes four axonal delay paths, the number of the inter-spike intervals that our neural network can store is simply equal to the number of axon modules. If, for instance, the patterns to be stored each contain 50 inter-spike intervals, the maximum number of such patterns that can be stored in the neural network is 82 (4k/51).

The patterns are trained only once when using delay programming. There is also only one recall test as there is no adaptation and the result of a recall will be the same each time. For each configuration of the neural network, 10 test runs were conducted. Different patterns are generated for different configurations as the LFSR that is used to generate the neuron index will use different polynomials for neuron arrays with different sizes (e.g., a 7-bit polynomials for the neuron array with 128 neurons, an 8 bit one for the neuron array with 256 neurons and so on). However, the LFSR used to generate inter-spike intervals will use the same polynomial for all the configurations and we use the same 10 random seeds for each configuration. This means that the patterns generated by the same seed will have the same random inter-spike intervals, which ensures the results obtained will fairly show the effect of different neuron array sizes.

**Figure 11:**
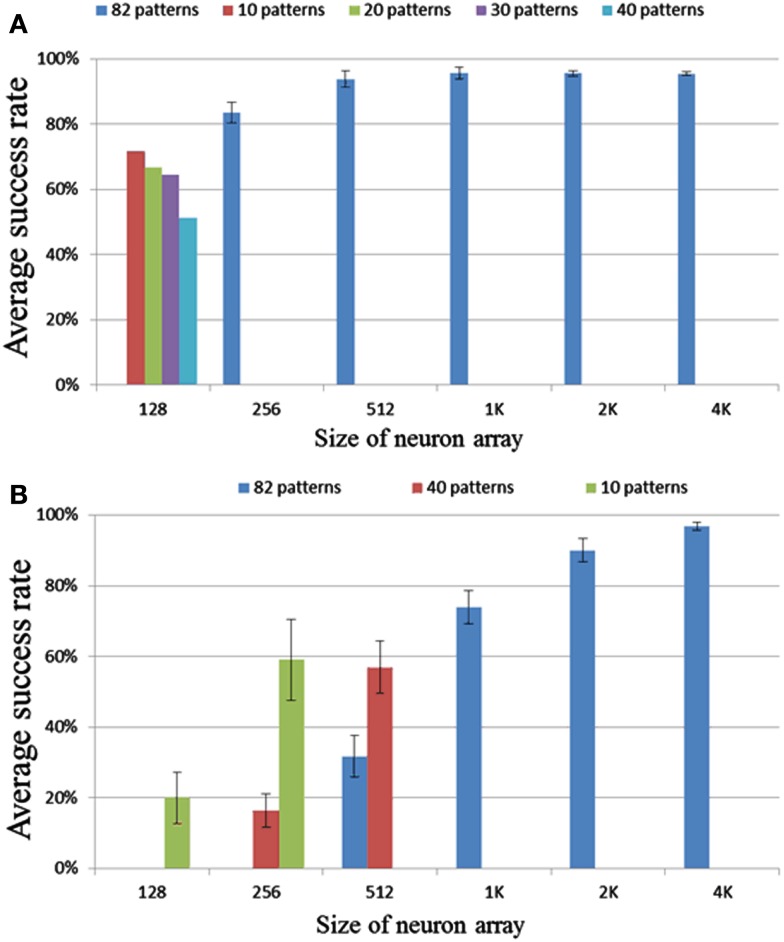
**Results for different neuron array sizes and pattern lengths using (A) delay programming and (B) delay adaptation**.

We tested neuron array sizes from 128 to 4k neurons. No noise was added in this set of tests. In the range of 256 to 4k neurons, each run had 82 patterns with a pattern length of 50 intervals. Figure 11A shows the results or these tests. The error bar is the standard deviation over 10 runs. It is clear that for a size larger than 256 neurons, the success rate is greater than 90%, and for a size of 256 neurons the success rate is above 80%.

For a configuration consisting of 128 neurons, the neural network will enter a state in which all the neurons fire simultaneously during recall if trained with 82 patterns. This is an extreme case of cross-talk. Cross-talk occurs when a neuron belonging to one pattern fires accidentally as a result of pre-synaptic spikes from other patterns or another part of the same pattern. The effect of the cross-talk depends on the overlap (correlation) of the patterns and can be regarded as noise. The more overlap there is, the higher possibility that a pattern plus some noise spikes will also set-off a different pattern. Also, the more input connections a neuron has, i.e., the more patterns this neuron is a member of, the more likely this neuron is to get three simultaneous inputs as a result of noise. To mitigate this problem, we need to increase the sparsity of the neural network, i.e., decrease the number of patterns a neuron is part of. This can be achieved by increasing the size of the neuron array as the patterns generated by the pattern generator are evenly distributed over the whole network. For a neuron array with 256 neurons, which is able to recall most stored patterns successfully (Figure 11A), in order to store 16K variables, each neuron will need to have an average of 64 (16K/256) connections – essentially 1/4 the size of the neuron array. Our measurements show that when the average number of connection for each neuron is smaller than 1/4 the size of the neuron array, cross-talk is not much of an issue.

The above results are for patterns with 50 inter-spike intervals. We have conducted the same tests with pattern length ranges from 20 to 128 inter-spike intervals and the results showed slight differences. The successful recall percentage for 20 inter-spike intervals is slightly better and for 128 intervals is slightly worse. This makes sense as the shorter the pattern is the more likely it will be recalled successfully and vice versa. To give a fair representation of the performance of the network, we are showing the results for patterns with 50 inter-spike intervals, which present an average performance.

### Delay adaptation

In the tests for the delay-adaptation mode (see Figure 11B), each pattern was trained five times and recalled one time. The strategy used adapted the delay by half the time difference between the pre- and post-synaptic spikes each time a neuron fired. The same settings used in the delay programming scenario were used for these tests.

The results for the smaller networks, of 128, 256, and 512 neurons, are much worse in this mode, than their counterparts in the delay-programming mode. The result for 1k neurons is close to the result of 256 neurons in delay-programming mode. The result for 2k and 4k neurons are similar to their counterparts in Figure 11A. These results suggest that cross-talk is a more serious problem when using delay adaptation. In delay-programming mode, a new pattern trained will not affect the patterns trained previously, but when using delay adaptation, new patterns will affect previously trained ones by adapting their delays, leading to increased cross-talk. To mitigate this problem, as explained in Section “Delay Programming,” we need to decrease the number of patterns a neuron is part of by increasing the size of the neuron array. To mitigate this problem at least 1k neurons are needed, so that the average number of connections per neuron is 16 or less.

### Characterization of pattern recall

In previous section we have presented the result in the setting that if more than 70% of spikes in a pattern have been recalled, we define this pattern as having been recalled successfully. In order to quantify the effect of selecting this arbitrary threshold, we have performed a test in which we count exactly how many spikes were correctly recalled in each pattern. All other settings were kept the same as used previously in the delay-programming mode and the delay-adaptation mode.

**Figure 12:**
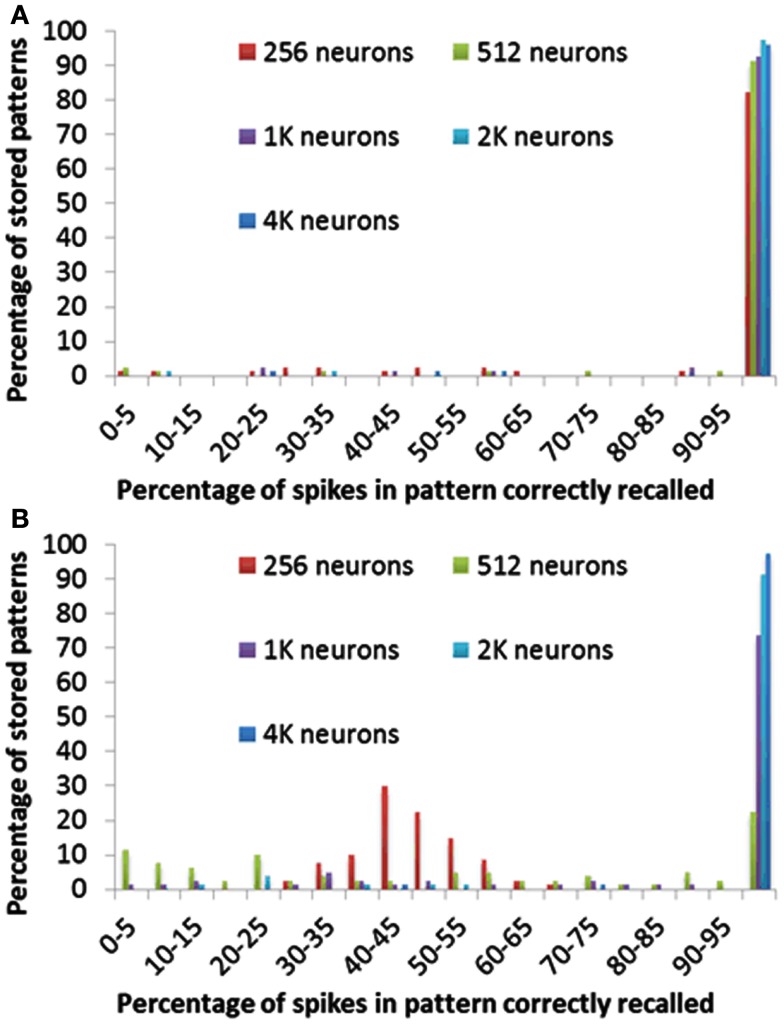
**Percentage of spikes in pattern correctly recalled for different neuron array sizes using (A) delay programming and (B) delay adaptation**.

Figure 12 shows for each pattern stored in the array how many spikes were recalled correctly. Figure 12A clearly indicates that more than 95% of the spikes are correctly recalled for most patterns in all configurations of the delay-programming mode. Figure 12B shows, as noted in the previous section, that smaller networks, i.e., networks with more patterns per neuron, perform much more poorly in the delay-adaptation mode. The networks with 2k and 4k neurons, however, recall more than 95% of the spikes correctly in more than 92% of all stored patterns.

### Effect of noise

In this set of tests, random noise was injected into the network. The Poisson rate of the noise, generated by the LFSR (see Pattern Generator and Checker), was varied from 2 to 128 spikes per second. This firing rate represents the number of additional spikes, i.e., not belonging to any of the trained patterns, presented to the network in a 1 s window. As each spike is generated by a randomly chosen neuron, the spike rate measures the total noise input, not the firing rate of individual neurons. For comparison, the firing rate of a stored pattern is about 100 spikes per second (50 events in about 500 ms).

**Figure 13:**
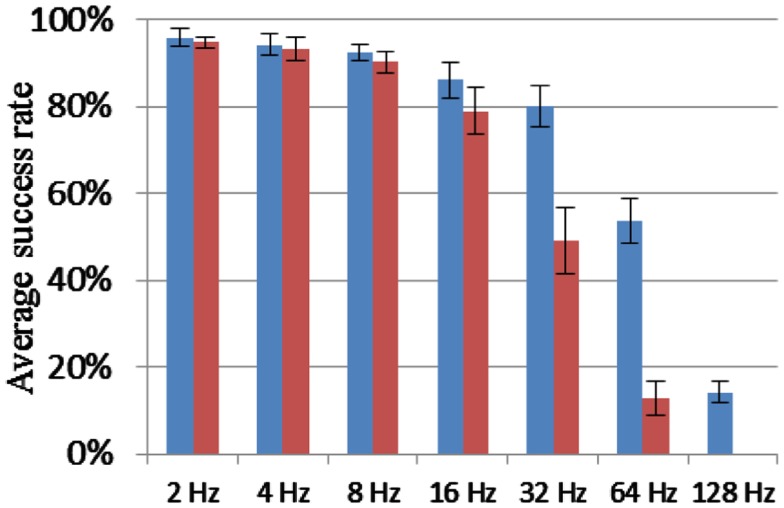
**Recall percentage for various Poisson rates of the noise generator**. Blue columns: delay programming; Red columns: delay adaptation.

All the other settings were kept the same as used in the delay-programming mode and the delay-adaptation mode with a neuron array consisting of 4k neurons. In both modes, no noise was added during the first training time. Figure 13 shows that the system is fairly robust to the noise when the Poisson rate of the noise is smaller than 32 and 16 Hz, respectively, in the delay-programming mode and delay-adaptation mode.

### FPGA capacity testing

**Figure 14:**
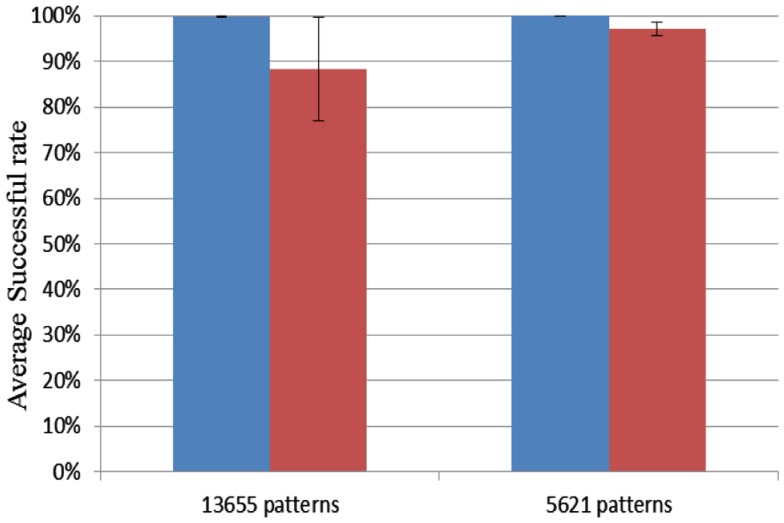
**Result for FPGA capacity testing**. Blue columns: delay programming; Red columns: delay adaptation.

To test the capacity of the Xilinx Virtex 6 XC6VLX240T evaluation board we used up all the hardware blocks, yielding one block of 4k neurons and 70 blocks of 4k axon modules for a total of 1.15M axons. Delay programming and delay adaptation were both used with pattern lengths of 21 and 51 spikes. The patterns were trained and recalled once. For a pattern length of 51 spikes, the maximum number of patterns that this configuration can store is 5621. Similarly, for a pattern length of 21 spikes, this number increases to 13653. For each pattern length, 10 test runs were conducted. Ten random seeds were used for each length. Figure 14 shows that the successful recall rate is higher than 96% on average for the four different scenarios. It proves that the proposed neural network is capable of operating successfully when all the axonal delay paths (variables) in the neural network are used. Each neuron receives on average 280 connections in this configuration.

## Discussion

Our implementation differs from the polychronous network introduced in Izhikevich (2006) in several important aspects. In Izhikevich (2006) a fully connected network is created with random delays, and STDP is used to prune the connections. Patterns are not stored or programmed into the network, but rather, random patterns emerge. A single connection between neurons could be active in a number of patterns, while other connections will become totally inactive. Izhikevich observes that “the number of co-existing polychronous groups could be far greater than the number of neurons in the network, sometimes even greater than the number of synapses.”

In our implementation, patterns can be directly programmed into the network, effectively creating a memory for spatio-temporal patterns that can be recalled by presenting the first four spikes in the pattern to the network. An additional advantage is that no connections remain unused when the maximum number of patterns has been programmed into the network. We aimed to avoid inactive connections, since hardware on the FPGA would still be dedicated to these inactive connections, but never used.

Each spike in the spatio-temporal pattern is generated by a neuron with delayed inputs from the previous four neurons in the pattern. In other words, four connections are needed for each spike in the pattern (except for the first four spikes that start the pattern). Since each neuron module contains four axons, the maximum number of spike intervals that can be stored in our network is simply equivalent to the number of axon modules on the FPGA. An additional constraint is that the number of patterns per neuron is small so that the patterns do not overlap excessively, which would cause too much cross-talk and poor pattern recall. Our experiments indicate that if the average number of connections per neuron is kept below 1/4 of the number of neurons, cross-talk is not much of an issue.

It is possible that more patterns could be stored in our hardware implementation if we allowed a connection to be active in more than one pattern, i.e., if we actively looked for correlation between patterns to be stored and reused parts of patterns that have already been stored. This would result in a significantly more complex pattern programming routine. It seems likely too, that in such an implementation, cross-talk would be a larger issue, so that the potential benefits of reusing connections would need careful investigation.

The test results also show that four times more neurons are needed for the same number of connections to obtain identical performance using the delay adaptation as opposed to delay programming. This raises the question as to why one would use delay adaptation at all. If an application requires storage of spatio-temporal patterns on an FPGA, then indeed, delay programming should be favored. Delay adaptation allows a network that is configured with random delays, or possibly programmed with a set of delays, to adapt to repeated patterns presented to it. So if, for instance, a spatio-temporal pattern slowly changes over time, the delay adaptation version of the network would track these changes, while the delay programming one would not. Furthermore, in less precise systems, such as analog VLSI implementations, it will be less likely that delay programming would get each delay exactly correct and delay adaptation will be needed to fine-tune the delays. Our experiments show that a network using delay adaptation will work as long as the number of patterns per neuron is kept sufficiently small; around eight times fewer connections per neuron should be used in the delay-adaptation mode than in the delay programming mode.

## Conclusion

We have presented an FPGA implementation of a polychronous spiking neural network with programmable delays. Time multiplexing was used to implement up to 1.15M axons, which could be fully utilized to store spatial-temporal patterns. To handle the high firing rate of the pre-synaptic spikes generated by these axons, a multiplexed neuron array with 4k virtual neurons was implemented by using 128 physical neurons. The test results show that, on average, the proposed neural network is capable of successfully recalling more than 95% of the spikes for 96% of the patterns stored in the network when the network is fully utilized, i.e., the maximum number of patterns are stored in the network. The tests also showed that the neural network is robust to noise.

## Conflict of Interest Statement

The authors declare that the research was conducted in the absence of any commercial or financial relationships that could be construed as a potential conflict of interest.
